# Refining Salinivibrio pangenome dynamics and biotechnological potential through comparative analysis

**DOI:** 10.1099/mgen.0.001786

**Published:** 2026-07-20

**Authors:** Crystal E. Young, Harrison O'Sullivan, Hussain Alattas, Ravi Tiwari, Andrew Macrae, Daniel V. Murphy, Colin Scott

**Affiliations:** 1 Bioplastics Innovation Hub, Food Futures Institute, Murdoch University, Murdoch, Western Australia, Australia; 2 School of Medical, Molecular and Forensic Sciences, Murdoch University, Murdoch, Western Australia, Australia; 3 Institute of Microbiology, IMPG, Universidade Federal do Rio de Janeiro, Rio de Janeiro, Brazil; 4 CSIRO Environment, Canberra, ACT, 2601, Australia

**Keywords:** biotechnology, extremophiles, hypersaline environments, pangenome

## Abstract

Current understanding of genomic diversity within the halophilic genus *Salinivibrio* relies predominantly on draft genomes, with only seven complete genomes among the 62 publicly available. Previous pangenome analysis suggested a closed genomic structure while concluding that *Salinivibrio* lacks polyhydroxyalkanoate (PHA) degradation capacity despite possessing biosynthesis genes. Here, we present eight complete *Salinivibrio* genomes from Pearse Lakes (Rottnest Island, Western Australia) generated using Oxford Nanopore long-read sequencing, alongside re-analysis of 38 high-quality public genomes (≥90% completeness and ≤5% contamination cut-off). Pangenome analysis revealed a more open structure than previously reported, with a core genome comprising 25% of total gene clusters and an accessory genome accounting for 71%. Panstripe analysis demonstrated significant temporal signal in gene gain and loss events associated with phylogenetic branch length (core: *P*=1.72×10⁻⁴; tip: *P*=2.64×10⁻¹⁴). All 46 genomes contained complete PHA biosynthesis operons (*phaB-phaA-phaP-phaC*) with high sequence conservation under strong purifying selection (Z=30.30, *P*<0.001). In a genome that readily gains and loses genes, this conservation indicates that PHA synthesis is a maintained pathway, which is difficult to reconcile with a previous report that *Salinivibrio* lacks PHA degradation capacity. We therefore searched the genomes by Hidden Markov Model-based homology rather than standard annotation and identified seven putative depolymerases that form a single accessory cluster in 15% of strains, all previously annotated as 3-oxoadipate enol-lactonase-2. These candidates retained all catalytic residues characteristic of active depolymerases but are divergent from reference PHA depolymerases which could explain why annotation missed them. They remain putative and require biochemical confirmation. Both the expanded pangenome and these candidates emerged from standardized homology-based re-analysis, showing that annotation-dependent approaches can overlook genomic diversity and divergent enzyme families in non-model organisms. Together, these results establish *Salinivibrio* as a genomically dynamic genus with potential for halophilic bioplastic production.

**Impact Statement**

Pangenome analyses of non-model bacteria are often constrained by the fragmented nature of the draft assemblies available. These fragmented assemblies can lead to misleading signals of genomic closure. Here, we present eight complete genomes from an underexplored hypersaline system. This study demonstrates that the *Salinivibrio* pangenome is open with a significant temporal signal in gene exchange, contrasting with previous reports of pangenome saturation. Furthermore, the identification of putative polyhydroxyalkanoate depolymerases, initially misannotated, highlights how some annotation approaches can miss homologs while providing novel targets for future enzyme characterization. These findings are relevant to halophilic genome evolution and the development of *Salinivibrio* as a chassis for bioplastic production.

## Data availability

All genomic data used in this study have been submitted to NCBI under project accession number PRJNA1039979. Other publicly available (NCBI) *Salinivibrio* genomes used in this study are listed in Table S1.

## Introduction

Hypersaline environments, both natural (e.g. hypersaline lakes, halite deposits, brines and salt flats) and anthropogenic (e.g. salterns, salt wells and salt fields), support microbiomes that are subjected to extreme conditions [1,2]. Such environments host microbiota adapted to grow with fluctuations in metal ions, nutrient availability, oxygen, pH, temperature and solar radiation [1]. While draft genome sequencing has enhanced our understanding of microbial diversity in these environments, complete genome sequencing remains fundamental for accurate insights into genome architecture and pangenome dynamics [3]. The genus *Salinivibrio,* where genomic diversity has been assessed largely from draft assemblies, has emerged as a valuable model for studying bacterial adaptation to moderately halophilic conditions, producing biotechnologically relevant enzymes (including chitinases, azoreductases, extracellular proteases, DNases, carboxylases and lipases) and biopolymers [4,12]. Moreover, *Salinivibrio* species are commonly isolated from hypersaline and saline environments because they grow rapidly on rich commonplace laboratory media [13]. This rapid growth characteristic also suggests that *Salinivibrio* could be an ideal chassis for various biotechnological applications where natural fast-growing micro-organisms are advantageous [14].

*Salinivibrio* (initially isolated in Australia and classified as *Vibrio*) belongs to the family Vibrionaceae within the class Gamma-proteobacteria [15,17]. Since the reclassification of *Salinivibrio*, six validly described species have been identified: *Salinivibrio costicola* (including subspecies *alcaliphilus*), *Salinivibrio proteolyticus* (including former subspecies *vallismortis*), *Salinivibrio sharmensis*, *Salinivibrio siamensis*, *Salinivibrio socompensis* and *Salinivibrio kushneri* [18,23]. *Salinivibrio* are Gram-negative, rod-shaped, non-spore-forming facultative anaerobes that have been isolated from a range of high-salt environments, including fermented foods, marine waters, salt wells, salterns and hypersaline lakes [18,19].

Among these high-salt environments, hypersaline lakes that have undergone transitions in their history can provide unique insights into microbial adaptation [24]. The hypersaline lakes on Rottnest Island, positioned ~30.4 km offshore from Perth, Western Australia (WA), exemplify such systems. These hypersaline lakes formed following sea-level changes between 7,000 and 5,000 years ago, when Rottnest Island became isolated from the mainland [25]. Like other isolated hypersaline systems (e.g. Lake Hillier, WA) that lack direct marine reinoculation, Pearse Lakes develop salt crusts in late summer when they dry out completely [25,26]. These fluctuating conditions may select for distinct genomic adaptations not captured in previous studies of more stable hypersaline environments [25,27].

These adaptations remain poorly resolved, as current understanding of *Salinivibrio* genomic diversity relies primarily on draft genomes, with only seven complete genomes among the 62 available in NCBI [13]. A previous study investigated orthologous gene clusters to define the *Salinivibrio* pangenome, including the assembly of the first complete genome of *S. kushneri* AL184^T^ [13]. From these results, the progression of the pangenome followed an asymptotic curve, with saturation observed after analysis of 38 genomes [13], indicating a relatively ‘closed’ pangenome. Underexplored niches like Pearse Lakes, however, may reveal strains with an expanded accessory genome encoding distinct adaptations [28,29]. The same reliance on draft genomes can also obscure metabolic functions, including pathways for polyhydroxyalkanoate (PHA) metabolism, that annotation-dependent approaches fail to detect in non-model organisms. We therefore hypothesized that additional complete genomes from underexplored environments could both expand the known pangenome and resolve metabolic functions that the draft genomes available to earlier analyses could not capture [3,30].

In this study, we present eight new complete *Salinivibrio* genomes from Pearse Lakes (Rottnest Island, WA) generated using Oxford Nanopore long-read sequencing, alongside a comprehensive re-analysis of 38 high-quality public genomes (≥90% completeness, ≤5% contamination). We tested three hypotheses: [1] complete genome assemblies from underexplored hypersaline environments would reveal genomic diversity not captured by draft assemblies [2]; the *Salinivibrio* pangenome would exhibit ongoing gene exchange rather than asymptotic closure, while core biosynthetic pathways, particularly PHA biosynthesis, would remain functionally constrained under purifying selection; and [3] comprehensive Hidden Markov Model (HMM)-based searches would identify metabolic genes missed by annotation-dependent approaches.

## Methods

### Sample site isolation and cultivation conditions

*Salinivibrio* spp. strains were isolated from Pearse Lakes, Rottnest Island (Wadjemup), off the coast of Perth (Boorloo), WA. Surface water samples were collected from a single site at Pearse Lakes (S 32° 0′ 22.281′ E 115° 30′ 44.484′) in May 2022, following the procedure described previously [31,33]. Briefly, 1,500 ml of water was collected and stored at 4 °C before processing. For physicochemical analysis, four independent surface water samples were also collected from the same site in separate bottles. These samples were filtered through a 0.2 µm Nalgene membrane filtration apparatus (Thermo Fisher Scientific, USA) and sent to the Marine and Freshwater Research Laboratory (Murdoch University, WA) (Accreditation Number: 10603) for physicochemical analysis.

Micro-organisms from the stored samples were cultivated in either buffered isolation medium (IM) or buffered lysogeny broth (LB). These media were chosen because Salinivibrio strains readily grow on rich media when supplemented with appropriate salt concentrations [13]. Buffered IM broth contained (per litre): 10.0 g Bacto-tryptone, 5.0 g Bacto-yeast extract, 2.0 g KCl, 20.0 g MgSO_4_ and 2.4 g HEPES (pH 7.8), adapted from Singh and Singh [34]. Buffered LB contained (per litre): 10.0 g Bacto-Tryptone, 5.0 g Bacto-yeast extract and 2.4 g HEPES (pH 7.8), adapted from Lennox [35]. Media were supplemented with 150 g l^−1^ NaCl, and the pH was adjusted to 7.8 with 5 M NaOH unless otherwise stated. Broths were cultured at 25 °C on an incubated shaker (Φ70 mm) at 200 r.p.m. Agar was incorporated at 1.5% (wt/v) if solid media were required. Single colonies of each isolate were serially re-streaked between the alternate media to ensure each isolate was singularly consistent and pure. Pure isolates were stored at −80 °C in 15% (v/v) glycerol.

### 16S rRNA gene analysis for *Salinivibrio* isolate identification

Single colonies of a pure culture were resuspended in 100 µl of PrepMan™ buffer (Applied Biosystems, Thermo Fisher Scientific, USA), vortexed and heated to 100 °C for 10 min. 16S rRNA V1–V4 regions were sequenced at the Australian Genome Research Facility. Sequences were aligned to the NCBI 16S rRNA type strain database (https://www.ncbi.nlm.nih.gov/) using blastn. The closest matched sequences were trimmed using Geneious Prime (v2024.0.5) and aligned using muscle (v3.8.425) using default parameters [36]. The best-fit model was selected in mega11 (v11.0.13), which selected the Tamura 3-parameter model with a gamma distribution and invariant sites (T92+G+I). The phylogeny was inferred by maximum likelihood with 100 bootstrap replicates, and the final tree was edited with ITOL (v6.0) and Inkscape [v1.3.2; (https://inkscape.org/)] without altering topology or statistical relationships [37,40].

### Genome DNA extraction and whole genome sequencing

Genomic DNA from the Pearse Lakes’ *Salinivibrio* spp. isolates were extracted using the cetyltrimethylammonium bromide (CTAB) extraction method [41]. Extracted genomic DNA was resuspended in 100 µl of nuclease-free water and stored at −20 °C. The quality and concentration of the extracted DNA were determined by spectrophotometry using a Beckman DU-640 Spectrometer (Beckman, USA). The concentration of DNA yielded for each isolate ranged from 1,000 to 5,000 ng µl^−1^, with a ratio (260/280) range of 1.80–1.90.

### Genomic sequencing and read quality check

Purified genomic DNA from *Salinivibrio* spp. isolates were used to prepare an Oxford Nanopore Technology (ONT) library according to the manufacturer’s protocol (https://nanoporetech.com/documentation). Sequencing was performed on a PromethION 2 (P2) using FLO-PRO114M flow cell (R10.4.1 chemistry) and the ONT native barcoding kit (Q20+) (SQK-NBD114.24). MinKNOW GUI software (v23.11.11) containing Dorado (v0.7.0) was used for basecalling and demultiplexing with adaptor trimming as a selected parameter (https://github.com/nanoporetech/dorado). The resulting long-read sequence data was assessed with NanoPlot (https://github.com/wdecoster/NanoPlot.git) (v1.41.6) with default parameters [42].

### Genome assembly and quality check

ONT long reads were filtered using NanoFilt (v2.8.0) (https://github.com/wdecoster/nanofilt.git) with selected parameters: (1) minimum read length 1,500 bp and (2) minimum Q score of nine [42]. *De novo* assembly of *Salinivibrio* spp. isolates was performed with Flye (v2.9.2) using default parameters with nine iterations to optimize assembly accuracy and contiguity, particularly in repetitive regions [43,44]. Assembly statistics were evaluated using the programme ‘anvi-display-contig-stats’ Anvi'o (v8.0 [45]). The quality of the completed assemblies was evaluated with CheckM2 (v1.0.2 [46]) and Benchmarking Universal Single-Copy Orthologs (BUSCO) (v5.6.1 [47,48]).

### Phylogenetic analysis

Average nucleotide identity (ANI) was used to analyse the genetic relatedness among *Salinivibrio* spp. analysed. The ANI-blast (ANIb) algorithm (blast+v2.11.0) was implemented using pyANI (v0.2.12) [45] with a >95% threshold for species identification [49]. ANIb percentage identity was visualized using a heatmap created with the ComplexHeatmap package (v2.13.1 [50]) in R-Studio (v4.3.0). Digital DNA–DNA hybridization (dDDH) was performed using the type (strain) genome server workflow, with dDDH score >70% indicating the same species, based on overall genomic similarity [51]. Multi-locus sequence analysis (MLSA) was conducted using AutoMLST (v7.0 [48]) to gain insight into the evolutionary history and genetic mechanisms of speciation. *Grimontia hollisae* ATCC 33564^T^ was used as the outgroup, as this species belongs to Vibrionaceae but lies outside the genus *Salinivibrio*, representing a closely related lineage within the *Salinivibrio–Grimontia–Enterovibrio* cluster [52]. The resulting phylogenetic tree was edited with iTOL and Inkscape, incorporating emended names previously proposed [13,18, 39].

### *Salinivibrio* pangenome analysis and post-analysis

The eight *Salinivibrio* strains were compared against 38 genomes from NCBI GenBank (≥90% completeness and ≤5% contamination cut-off) following established standards for high-quality bacterial genomic assemblies [53,54]. *S. socompensis* S10B (GCF_000565325.1) and *S. socompensis* S35 (GCF_000513715.1) both failed to meet the cut-off; however, they were included as they were the only representative species available. Metadata (e.g. isolation location) was collected for the NCBI strains (Table S1, available in the online Supplementary Material). To minimize artefacts from assembly fragmentation, we prioritized complete and near-complete genomes and applied standardized re-annotation using Anvi'o (v8), with HMM and Prodigal (v2.6.3 [55]) to ensure consistent gene calling across all 46 genomes. Functional annotations were assigned with clusters of orthologous groups (COG20; 2020 release) and Kyoto Encyclopedia of Genes and Genomes (KEGG) orthologues using Anvi'o (Anvi'o tutorials, https://anvio.org/) [45]. The annotated genomes were exported from Anvi'o in GenBank format, preserving all functional annotations.

Pangenome analysis was performed in Anvi'o, using Markov cluster algorithm (MCL) for clustering and DIAMOND (--sensitive) (v2.1.8 [56]) for amino acid sequence similarity. Partial gene calls were not included in the analysis, and the remaining parameters required to define gene clusters according to amino acid sequence homology were: --minbit 0.5, --mcl-inflation 10, --min-percent-identity 0 and --min-occurrence 1. Briefly, the subsequent matched protein clusters were refined using the minbit heuristic, as previously defined [57]. Cluster granularity and sensitivity were further refined by the default MCL inflation parameter (*n*=10), suitable for genus-level comparisons and preventing over-clustering of gene families [58]. Gene collections were classified as core (gene cluster present in 100% of genomes), softcore (>95%), dispensable (≥2 genomes, <95%) and singletons (present in one genome) [29]. Resulting protein clusters were visualized using ‘anvi-display-pan’ and sorted by a ‘presence or absence’ scheme. The resulting figure was completed in Inkscape to adjust the font size and add a key on the left-hand side.

### Post-analysis of the *Salinivibrio* pangenome

Post-analysis of the *Salinivibrio* pangenome was also completed with the tool Panstripe (v0.3.0 [30]). In brief, the pangenome presence–absence matrix was exported from Anvi'o for inclusion in the analysis. Concatenated protein sequences of the core genome (1,172 single-copy core genes present in all 46 genomes) from Anvi'o were exported and aligned using MAFFT (v7.525 [59]) with the --auto parameter selected. MAFFT automatically selected the FFT-NS-2 algorithm and switched to memory-saving mode due to the dataset size. The resulting alignment was imported into the tool IQ-Tree (v2.3.6 [60]) with default parameters [60]. Model finder determined JTT+F+R10 to be chosen according to Bayesian information criterion (optimal log-likelihood, −1.76E+06). The resulting core phylogeny tree and presence/absence matrix were analysed using Panstripe (v0.3.0 [30]) using max parsimony as the method for ancestral-state reconstruction. Panstripe fits two parameters: ‘core’, which tests whether phylogenetic branch lengths are associated with gain and loss events and ‘tip’, which tests whether the rate of gene exchange at the tips of the phylogeny differs from the internal branches [30]. Panstripe analysis was visualized and edited for clarity with Inkscape.

### Estimated metabolic analysis of *Salinivibrio* spp.

Metabolic reconstruction with summaries of KEGG module-estimated pathway completeness (75% threshold) was performed in Anvi'o and visualized using ComplexHeatmap [45]. This threshold balances pathway-detection sensitivity with confidence, accounting for potential annotation gaps while maintaining reliability in pathway presence. COG20 category frequencies were visualized using ComplexHeatmap (RStudio package). Hierarchical clustering was performed using Euclidean distance and complete linkage for rows/columns using default parameters.

### PHA pathway analysis

PHA biosynthesis genes were identified from functional annotations obtained via Anvi'o, using KEGG ortholog assignments for acetyl-CoA acetyltransferase (*phaA*, K00626), acetoacetyl-CoA reductase (*phaB*, K00023) and PHA synthase (*phaC*, K03821); Phasin (*phaP*) was identified from PHA granule-associated annotations. Genomic regions flanking *phaC* (±10 kb) were extracted from all 46 genomes using custom Python scripts. Gene synteny was visualized using Clinker (v0.0.27 [61]) with default parameters. For each PHA biosynthesis gene (*phaP*, *phaB*, *phaA*, *phaC*), protein sequences were extracted from all 46 genomes and aligned using muscle (v3.8.1551 [36]), and pairwise amino acid identity, identity ranges and gene length were calculated using Geneious Prime (v2024.0.5). Purifying selection on PhaC was tested using the Nei-Gojobori method (HA: dN <dS, 100 bootstraps; 611 positions, pairwise deletion) in mega 11 (v11.0.13 [38,62]). A maximum likelihood tree was reconstructed from the codon-aligned sequences in IQ-TREE (TNe+G4, ModelFinder, 1,000 ultrafast bootstraps [63]).

### PhaZ depolymerase detection and validation

Putative PHA depolymerase genes were identified using an HMM-based approach. A custom HMM profile was constructed from 895 γ-proteobacterial PHA depolymerase sequences retrieved from UniProt [organism: ‘Gammaproteobacteria’ AND annotation: (type: function ‘PHA depolymerase’), downloaded November 2024]. No length filtering was applied, given the broad length range of bacterial PhaZ (92–1,060 aa). All protein-coding sequences from the 46 genomes (140,841 proteins) were searched against the profile using hmmsearch (E-value ≤1×10⁻⁵, bit score ≥30). The seven candidates were validated in Jalview (v2.11.3.2) for the GXSXGG lipase box and the conserved aspartate and histidine of the Ser-His-Asp catalytic triad [64]. Downstream PHA catabolic genes bdhA (K00019) and atoAD (K01907) were taken from KEGG annotations. To assess their affiliation with experimentally validated PhaZs, the candidates were aligned with 44 reference PhaZs (Table S1) using muscle v5.3 and placed in a maximum likelihood phylogeny tree to account for rate variation across these divergent sequences (IQ-TREE v2.3.6, LG+F+R8, 1,000 ultrafast bootstraps).

## Results and discussion

### Isolation of strains from Pearse Lakes, Rottnest Island, Western Australia

At the time of isolation, Pearse Lakes had a pH of 8.0±0.01 and salinity of 20.1±1.44% (mean±se, *n*=4). These conditions differ from general growth requirements for *Salinivibrio*, which grows optimally at NaCl concentrations between 2.5 and 10% (wt/v) and pH 7.3–7.8, but fall within broader tolerance ranges of 0–20% NaCl (wt/v) and pH 5–10 [8,15, 23, 65,68]. Hydrochemical analysis of Pearse Lakes indicates that the major solutes were total alkalinity (276.9±0.18 mg CaCO_3_ l^−1^), total acidity (50.1±2.11 mg CaCO_3_ l^−1^), dissolved organic carbon (63.3±0.96 mg C l^−1^), dissolved inorganic carbon (26.0±0.61 mg C l^−1^), total dissolved carbon (89.3±3.6 mg C l^−1^), total dissolved nitrogen (4.9±0.1 mg N l^−1^), ammonia (282.5±12.44 µg N l^−1^) and sulphates (SO_₄_^²⁻^; 14.0±0.0 g SO_4_^2-^ l^−1^). These measurements characterize the physicochemical conditions at the time of isolation and represent a single sampling event.

**Fig. 1.**
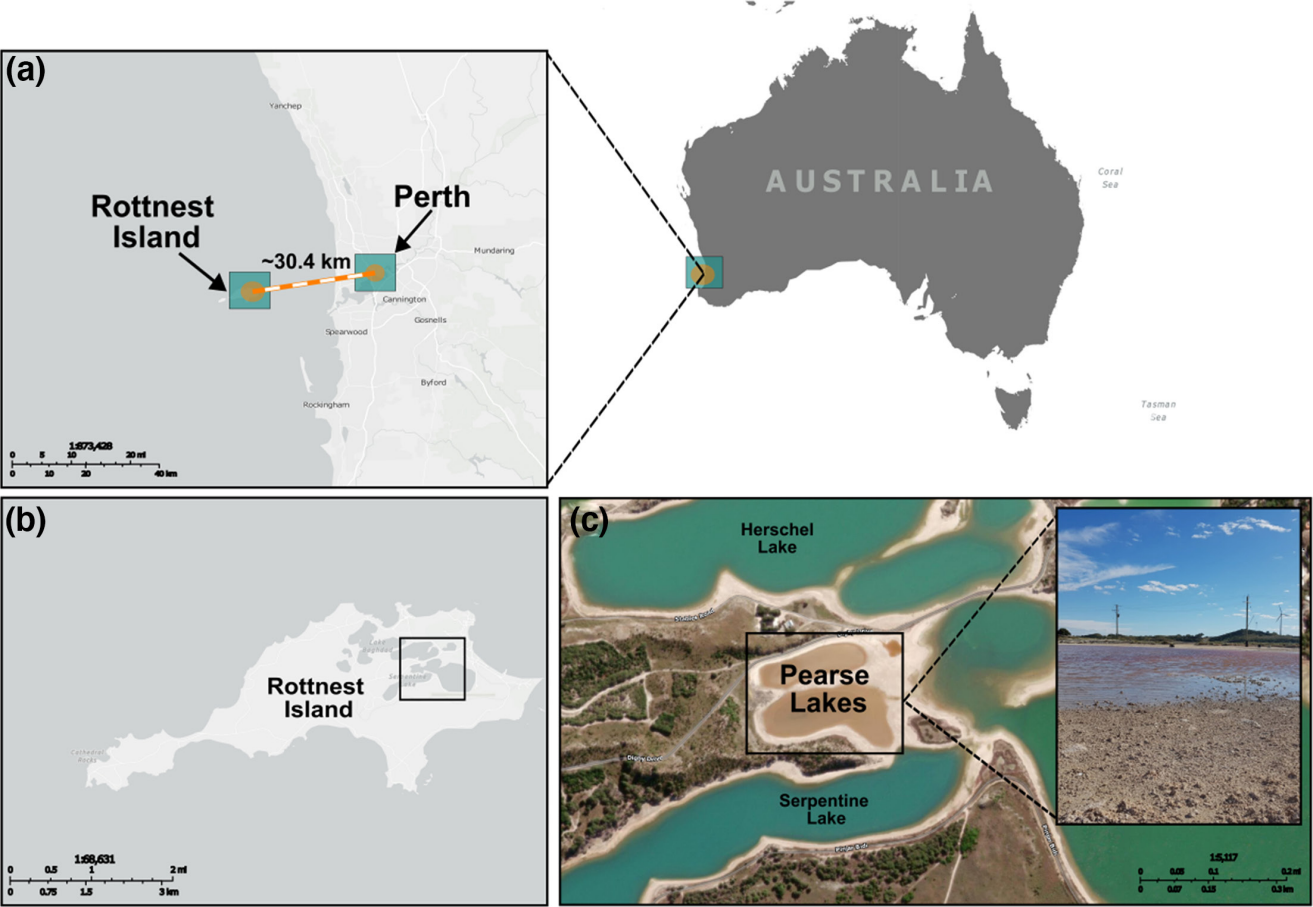
Study area and sampling site (S 32° 0′ 22.281′ E 115° 30′ 44.484′) at Pearse Lakes, Rottnest Island, WA. (**a**) Graphical map of Australia showing the location of Perth (teal-shaded region). (**b**) Graphical map showing the location of Rottnest Island, located ~30.4 km off the coast of Perth, WA. (**c**) Satellite/aerial image of the hypersaline Pearse Lakes (white box), with surrounding hypersaline lakes including Herschel, Government and Serpentine, located on Rottnest Island. (**d**) Photo taken (at time of collection) of the Pearse Lakes sampling site (S 32° 0′ 22.281′ E 115° 30′ 44.484′). Satellite images and graphical images were generated with ArcGIS-online (Esri Community Maps Contributors, OpenStreetMap, Microsoft, Esri, TomTom, Garmin, Foursquare, METI/NASA, USGS, Maxar). Images were exported from ArcGIS-online and edited in Inkscape to correct the font.

A total of 40 isolates were cultivated over a range of salinities (5–275 g l^−1^ NaCl), with eight of the isolates obtained showing growth phenotypes similar to those previously described for *Salinivibrio* [68] (Table S1). Members from the genus *Salinivibrio* have been previously isolated from a range of different environments, demonstrating their ability to adapt to varied NaCl concentrations and pH levels [21,22, 65,67]. Although the pH and salinity of Pearse Lakes (Fig. 1) are higher than the reported optimal ranges for *Salinivibrio*, they fall within the genus’s known tolerance limits, supporting the isolation of eight putative *Salinivibrio* strains. Pearse Lakes is also an ephemeral system that dries seasonally and forms salt crusts, so the isolates were obtained from a fluctuating rather than relatively stable hypersaline environment. Such variability is expected to favour broad physiological tolerance over narrow specialization, as organisms must persist across wide ranges of salinities rather than a single optimum, consistent with the broad tolerance reported for the genus [69].

### Initial identification via 16S rRNA sequencing for *Salinivibrio* strains

Eight of the 40 isolates obtained from Pearse Lakes were *Salinivibrio*, as demonstrated by phylogenetic analysis of the V1–V4 region of the 16S rRNA (Fig. S1). Initial identification of the different *Salinivibrio* species isolates compared with type strains showed that strains PL1-006, PL1-015, PL1-016 and PL1-017 shared common ancestry with *S. kushneri* AL184^T^. Strain PL1-002 grouped with *S. costicola* LMG 11651^T^, whereas PL1-003A, PL1-026 and PL1-028 clustered with *S. socompensis* S53. Given that *Salinivibrio* species typically share almost identical 16S rRNA sequences (1,209–1,470 bp) with 96.3–100% similarity, this marker lacks sufficient taxonomic resolution for reliable species delineation, as previously demonstrated [18]. Therefore, a complementary approach for taxonomic classification was employed: Overall Genome Relatedness Indices, including ANI and dDDH [54]. MLSA was conducted in parallel to improve phylogenetic characterization [18,54].

### Genomic characteristics and annotation summary of the *Salinivibrio* isolates

ONT long-read sequencing of eight *Salinivibrio* strains yielded complete assembled genomes ranging from 3,275,852 to 3,422,267 bp with a G+C content ranging from 49.29 to 50.86 mol% (Table 1). Coverage for the newly sequenced genomes ranged from 52× to 187× (Table 1). The *de novo* assembly of the Pearse Lakes strains resulted in each of PL1-002, PL1-003A, PL1-006, PL1-015, PL1-016, PL1-017, PL1-026 and PL1-028 yielding two circular contigs each with sizes ranging from 2.6 to 2.8 Mbp for contig 1 and from 550 to 630 Kbp for contig 2 (Table S1).

**Table 1.** Summary of *Salinivibrio* spp. Pearse Lakes isolates

Isolate name	No. of contig	Genome size	G+ C mol%	Mean coverage	CheckM2 completeness (%)	Contamination (%)	BUSCO score (ortholog set: Vibrionales)	CDS	rRNA	tRNA gene
**PL1-002**	2	3,302,407	49.39	71×	100	0.1	C:93.7% [S:93.6%, D:0.1%], F:1.2%, M:5.1%	3,000	9	95
**PL1-003A**	2	3,321,402	49.41	52×	100	0.86	C:92.8% [S:92.7%, D:0.1%], F:1.7%, M:5.5%	3,004	9	95
**PL1-006**	3	3,420,200	50.58	187×	99.91	1.1	C:95.2% [S:95.2%, D:0.0%], F:4.0%, M:0.8%	3,273	9	96
**PL1-015**	2	3,414,104	50.67	157×	99.99	0.07	C:97.6% [S:97.6%, D:0.0%], F:2.4%, M:0.0%	3,075	9	96
**PL1-016**	2	3,462,693	50.7	169×	100	3.02	C:95.8% [S:93.1%, D:2.7%], F:0.6%, M:3.6%	3,034	9	98
**PL1-017**	2	3,275,852	50.86	64×	100	0.04	C:95.3% [S:95.2%, D:0.1%], F:0.8%, M:3.9%	2,933	9	96
**PL1-026**	2	3,422,045	49.29	56×	99.99	1.56	C:93.5% [S:92.7%, D:0.8%], F:1.3%, M:5.2%	3,121	9	95
**PL1-028**	2	3,422,267	49.3	155×	100	0	C:94.8% [S:94.7%, D:0.1%], F:0.5%, M:4.7%	3,080	9	95

CDS, coding DNA sequences.

This bipartite genome organization aligns with the established architecture of the family of Vibrionaceae, in which most members have genomes consisting of a primary chromosome and at least one large secondary replicon (>350 Kbp) [70,71]. The secondary replicon, termed a chromid, in Vibrionaceae originates from plasmids that have coevolved with the primary chromosome and carry core cellular functions, mainly related to their maintenance in the genome [70,71]. Additionally, the functional role of chromids in Vibrionaceae is likely to be linked to environmental adaptation, with genes potentially associated with metabolism, stress response and niche-specific functions. Nevertheless, experimental validation is needed to test gene essentiality of the assembled chromids for the Pearse Lakes isolates [72].

A third circular contig was also assembled for strain PL1-006, suggesting that a small plasmid is also present (Table 1). A further inspection of annotations for putative replication genes and other indicators, such as putative toxin–antitoxin operons, agrees that the additional third contig assembled for these strains may be a plasmid. Other *Salinivibrio* isolates possessing small plasmids have been previously identified, with a comprehensive analysis, via pulsed-field gel electrophoresis, uncovering strain *S. costicola* E-367 with the smallest plasmid ~2.95 kb in size [73]. While our *in silico* analysis suggests the presence of a plasmid for PL1-006, additional validation of plasmid confirmation is needed [73,76].

Quality assessment for the Pearse Lakes assemblies was performed using both CheckM2 and BUSCO to investigate genome completeness and quality [46,48]. CheckM2 estimated completeness values ranging from 99.1 to 100% across all Pearse Lakes strains. While contamination values were generally low (0–1.56%), strain PL1-016 showed a higher value of 3.02%, which is still within acceptable thresholds for high-quality genomes according to the Minimum Information about a Single Amplified Genome standards [77]. These regions flagged as ‘contamination’ may represent strain-specific variations or halophilic adaptations, as quality assessment tools are primarily trained on non-halophilic reference genomes [46]. BUSCO analysis corroborated genome completeness, with complete (C) scores ranging from 92.8 to 97.6%, minimal duplication (D: 0.1–2.7%), low fragmentation (F: 0.5–4.0%) and few missing (M) genes (0–5.5%). The complementary application of these tools, despite their reference-based limitations for non-model organisms, confirms that our circular contigs represent complete, high-quality genome assemblies suitable for downstream analysis [77].

Following quality assessment, genome annotation was performed for the Pearse Lakes isolates. Genome annotations of the Pearse Lakes isolates predicted 2,989–3,273 coding DNA sequences (CDS) per genome. Furthermore, all isolates contained 9 rRNA operons and 95–98 tRNA genes (Table S1). Annotated CDS, rRNA operons and tRNA genes corresponded with previous work [13].

### Phylogenetic analysis of *Salinivibrio* isolates

#### Average nucleotide identity and digital DNA hybridization

**Fig. 2.**
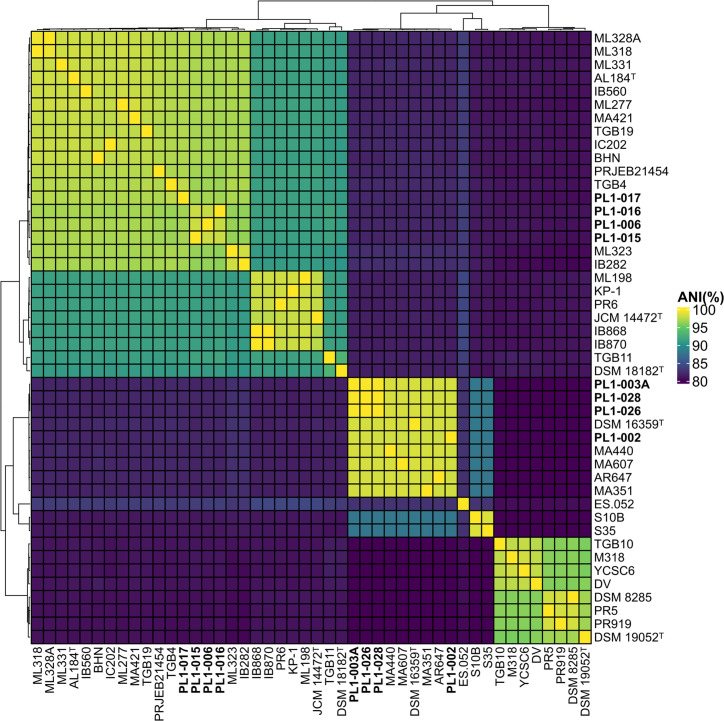
ANIb identity of *Salinivibrio* spp. isolates obtained from Pearse Lakes, Rottnest Island, WA, against selected *Salinivibrio* spp. genomes downloaded from NCBI (including type strains and non-type strains). ANI-blast (ANIb) algorithm (blast+2.110.0) was used via the software pyANI v0.2.12 with a threshold of 95–96% as the cut-off for species identification. A percentage identity matrix was used to create a heatmap representing ANIb clusters by the ComplexHeatmap package in R-Studio (Version 4.3.0). The exported heatmap generated from R Studio was edited with Inkscape (https://inkscape.org/) to correct the font size.

A comparison of ANI using blast (ANIb) was performed for the strains isolated from Pearse Lakes and for genomes collected from NCBI. ANIb analysis placed the Pearse Lakes isolates into two species: four strains (PL1-002, PL1-003A, PL1-026 and PL1-028) clustered with *S. costicola* (97.8–98.6%) and four strains (PL1-006, PL1-015, PL1-016 and PL1-017) clustered with *S. kushneri* (97.1–97.8%) (Fig. 2 and Table S1). Notably, PL1-017 clustered separately from the other *S. kushneri* isolates, showing closest affinity to *S. kushneri* TGB4 (97.77%). All values exceeded the 95–96% species boundary threshold [49,78]. Furthermore, dDDH was completed, which agreed that the current strains isolated from Pearse Lakes do not belong to any new species (Table S1) [51].

The strains isolated from Pearse Lakes were identified as *S. kushneri* and *S. costicola* using both ANIb and dDDH results. The high ANI values (97.13–98.64%) between Pearse Lakes isolates and strains analysed from geographically distinct locations suggest potential genomic conservation within these *Salinivibrio* species. In addition, no novel *Salinivibrio* species were identified, which may reflect the selection bias inherent to culture-dependent approaches, in which rich media favour fast-growing strains and potentially miss slower-growing or more fastidious species [79,80].

#### Multi-locus sequence analysis of *Salinivibrio* genomes

**Fig. 3.**
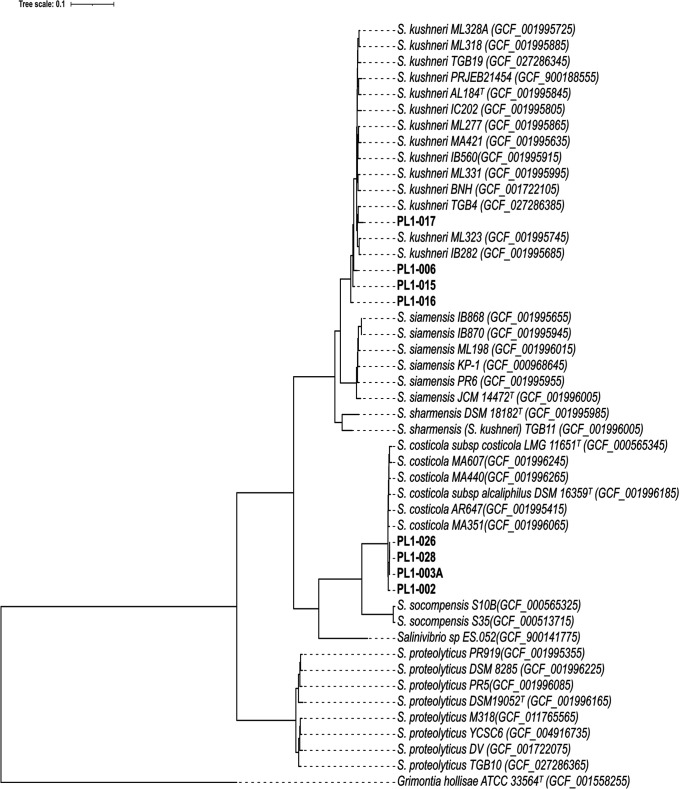
Maximum-likelihood genome-scale phylogenetic tree showing the evolutionary relationships of *Salinivibrio* strains isolated from Pearse Lakes, Rottnest Island, and *Salinivibrio* genomes collected from NCBI with amended names displayed [13]. Phylogeny is based on 81 gene sequences assembled using the Automated Multi-locus Species Tree (autoMLST) pipeline (https://automlst.ziemertlab.com) via concatenated alignment; the genes are listed in Table S1 [103]. Colours indicated in the legend indicate the percentage bootstrap values of 1,000 replicates (with 'Perform IQ-TREE Ultrafast Bootstrap analysis' option); only values >70% are indicated [60]. The tree generated was exported into Interactive Tree of Life (iTOL) for visualization [39] and edited with Inkscape (https://inkscape.org/) to correct colour and font size. The superscript T indicates type strains used. *G. hollisae* ATCC 33564^T^ was used as the outgroup, as selected by AutoMLST.

Phylogenetic analysis using MLSA resolved the 46 strains into six phylogroups (Fig. 3), consistent with recent literature [13,18, 19]. Pearse Lakes strains PL1-006, PL1-015, PL1-016 and PL1-017 grouped within a phylogroup alongside other *S. kushneri* strains, whereas PL1-002, PL1-003A, PL1-026 and PL1-028 were placed in a phylogroup with *S. costicola, S. costicola* subsp. *alcaliphilus* and *S. costicola* subsp. *costicola*. Notably, subspecies within the phylogroup were not resolved as distinct lineages from *S. costicola*. The extended dataset also highlighted unresolved taxonomy among publicly available genomes: *S. kushneri* TGB11 clustered within a phylogroup alongside *S. sharmensis* DSM 18182^T^, suggesting that its species assignment may require revision whereas *Salinivibrio* sp. ES.052 did not cluster within any phylogroup, consistent with its proposed status as a novel species [13].

### Pangenome analysis of *Salinivibrio* genomes

**Fig. 4.**
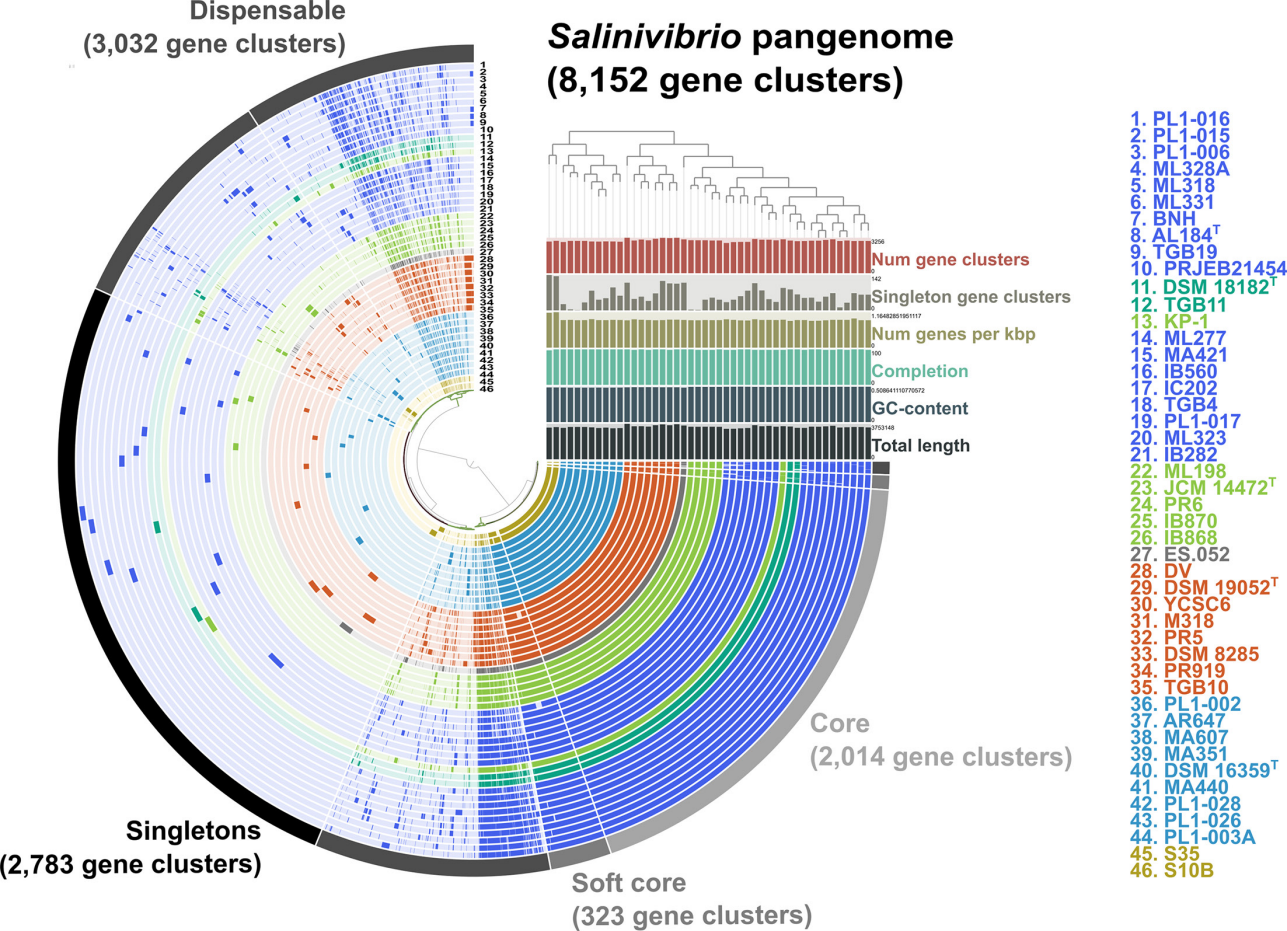
Pangenome of NCBI and Pearse Lakes' *Salinivibrio* strains isolated from varied hypersaline and saline environments generated with Anvi'o v8. Genomes are organized based on the tree of presence and absence of gene clusters (D: Euclidean; L: Ward) (Table S1). Each colour represents a different species as follows: *S. kushneri* (blue), *Salinivibrio* spp. (grey), *S. sharmensis* (dark green), *S. siamensis* (lime green), *S. proteolyticus* (orange), *S. costicola* (including *costicola* subsp. *alcaliphilus*) (aqua) and *S. socompensis* (gold). Outside highlights represent gene collections: core (gene clusters present in 100% of genomes), softcore (gene clusters present in ≥95% of genomes), dispensable (gene clusters present in at least two genomes and ≤95% of genomes) and singletons (singletons, genes present in just one unique genome). Additional information, such as total length, G+C content, completion, number of genes per kbp, singleton gene clusters and number of gene clusters, is represented by bars at the top right. The resulting image was edited with Inkscape (https://inkscape.org/) to adjust font size and add a key on the left-hand side of the figure.

The *Salinivibrio* protein-based pangenome (46 genomes) comprised 8,152 gene clusters (142,714 gene calls), of which 60% (4,824 gene clusters) had known COG functions (Fig. 4 and Table S1). More than two-thirds of the identified gene clusters had metabolic predictions (70%, 3,440 gene clusters with known KOs; 7%, 568 gene clusters with known KEGG class; Table S1). The core genome contained 2,014 gene clusters (25%; 95,122 gene calls) with an additional 323 softcore clusters (4%; 14,754 gene calls). The accessory genome is composed of 5,815 gene clusters (71%; 32,838 gene calls), consisting of 3,032 dispensable clusters (52% of accessory; 29,965 gene calls) and 2,783 singleton clusters (48% of accessory; 2,873 gene calls) (Fig. 4). In comparison, a previous first-genus-wide analysis of the *Salinivibrio* pangenome (45 genomes) identified 5,570 gene clusters, with 2,080 core (37.3%) and 3,490 accessory (62.7%) clusters [13], with differences likely attributable to genome quality and analytical approach as discussed below. A detailed list of genes, with each gene call cluster, putative function and homogeneity indices, is provided in Table S1.

### Post-analysis of the *Salinivibrio* pangenome

**Fig. 5.**
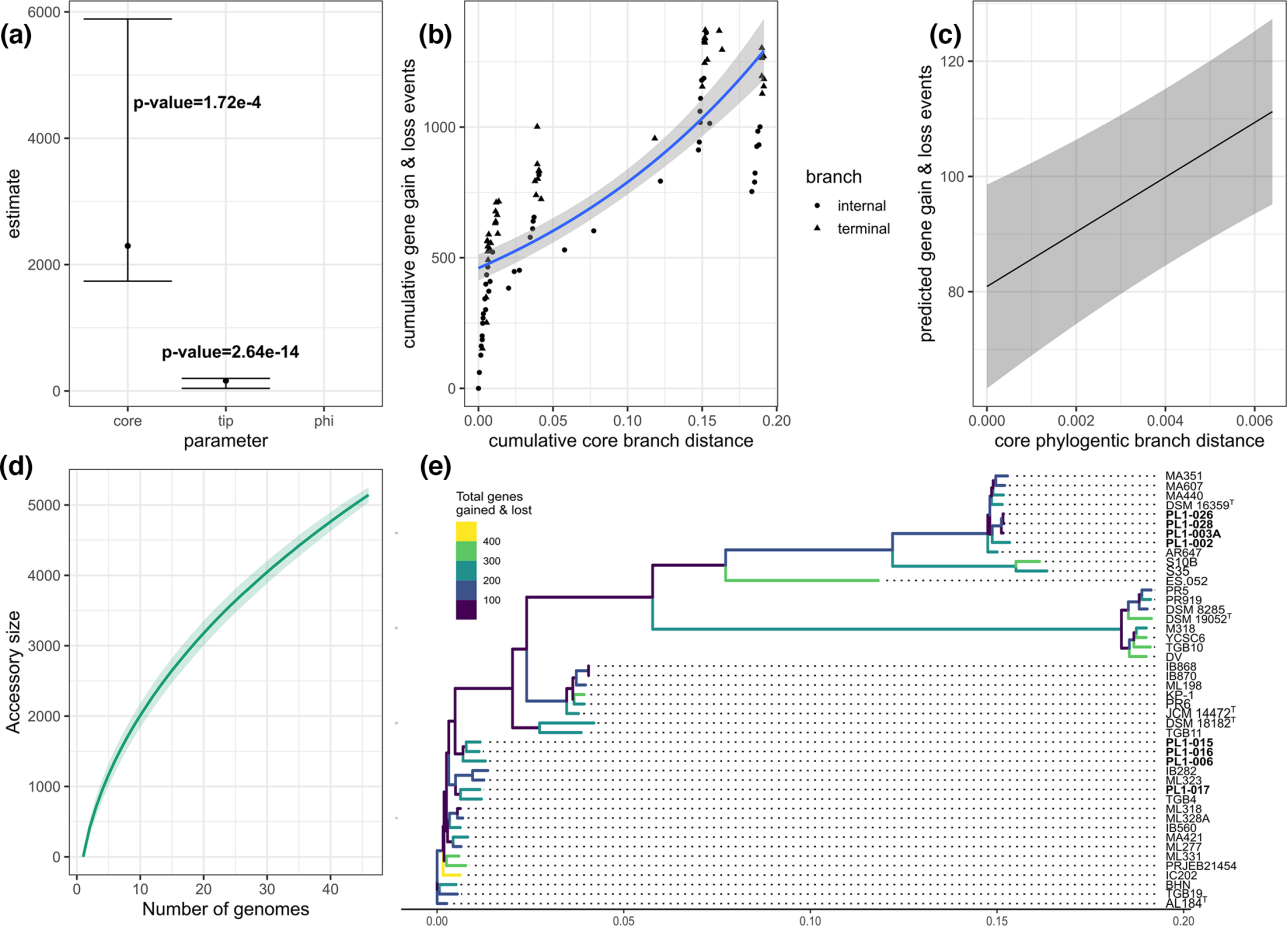
Post-analysis of the *Salinivibrio* pangenome analysis with Panstripe pipeline [30]. (**a**) Model estimate plot showing association between evolutionary time and gene exchange events (core: *P*-value=1.72e-4; tip: *P*=2.64e-14). (**b**) Empirical relationship between cumulative core branch distance and gene gain/loss events, with points distinguished by branch type. (**c**) Model predictions with 95% confidence intervals demonstrating the temporal signal in gene exchange. (**d**) Pangenome accumulation curve showing continued expansion without saturation. (**e**) Maximum likelihood phylogeny with branches coloured by inferred gene gain and loss events using maximum parsimony ancestral state. Analysis performed using Panstripe v0.3.0 in R v4.3.0 with a Gaussian family model and 1,000 bootstrap replicates. The resulting image was edited with Inkscape (https://inkscape.org/).

Pangenome results based on amino acid clustering were analysed using the Panstripe pipeline to determine the rate of gene exchange associated with branch length in the phylogeny (Fig. 5) [30]. Unlike the previously reported pangenome saturation, our accumulation curve does not approach asymptotic closure (Fig. 5d), suggesting ongoing expansion consistent with an ‘open’ pangenome [13]. However, accumulation curves alone do not account for the underlying population structure and are often influenced by genome sampling and annotation errors [3,30].

Panstripe modelling, which is robust to these biases [30], provided statistical support for an open pangenome. The core parameter indicated a significant association between phylogenetic branch length and the number of gene gain and loss events (*Salinivibrio* core: *P*-value=1.72e-04), meaning gene exchange accumulates proportionally with evolutionary divergence (Fig. 5a). The tip parameter showed that the rate of gene exchange at the tips of phylogeny differs significantly from internal branches (*Salinivibrio* tip: *P*-value=2.64e-14), indicating ongoing, recent gene flux (Fig. 5a) [29,30]. The distribution of ancestral gene gain and loss events at the tree’s tips further supports this interpretation (Fig. 5e). This pattern is consistent with ongoing gene exchange, and the geographic span of the dataset (Table S1) suggests this process occurs across diverse hypersaline environments [30].

The contrast with a previous analysis [13], which established the first genus-wide *Salinivibrio* pangenome framework, likely reflects differences in genome quality and analytical approach rather than fundamental disagreement. That study, constrained by the draft assemblies available at the time, identified 5,570 protein-based gene clusters from 45 mostly draft genomes, with the accumulation curve approaching saturation. Several factors differ between the analyses and cannot be separated in this current study. The previous study defined clusters using reciprocal best blast matches at 40% amino acid identity [13], whereas we used Markov clustering in Anvi'o; these algorithms group proteins differently and can yield different cluster numbers from the same input [3,30]. Gene calling also differed, with standardized re-annotation through Anvi'o with Prodigal and HMM-based gene calling across all 46 genomes, alongside the addition of eight completed genomes. Each of these factors can alter gene cluster counts, so the increase cannot be attributed to a single cause. The open pangenome conclusion, however, rests on the Panstripe modelling described above rather than on the absolute cluster count and therefore is supported independently of these differences [30,81,83]. These methodological advances, rather than contradicting the earlier findings, build on that foundation to reveal finer-scale pangenome dynamics within *Salinivibrio*. Nevertheless, limitations inherent to pangenome analysis remain, and emerging graph-based approaches may improve the consistency of gene prediction and clustering across populations, refining pangenome estimates [84]. Together, our findings reveal a dynamic genetic landscape within *Salinivibrio*, characterized by continuous gene acquisition and loss, highlighting the adaptive potential of this genus.

### Metabolism of *Salinivibrio* spp.

#### General gene ontology (COG20) for *Salinivibrio* genomes

**Fig. 6.**
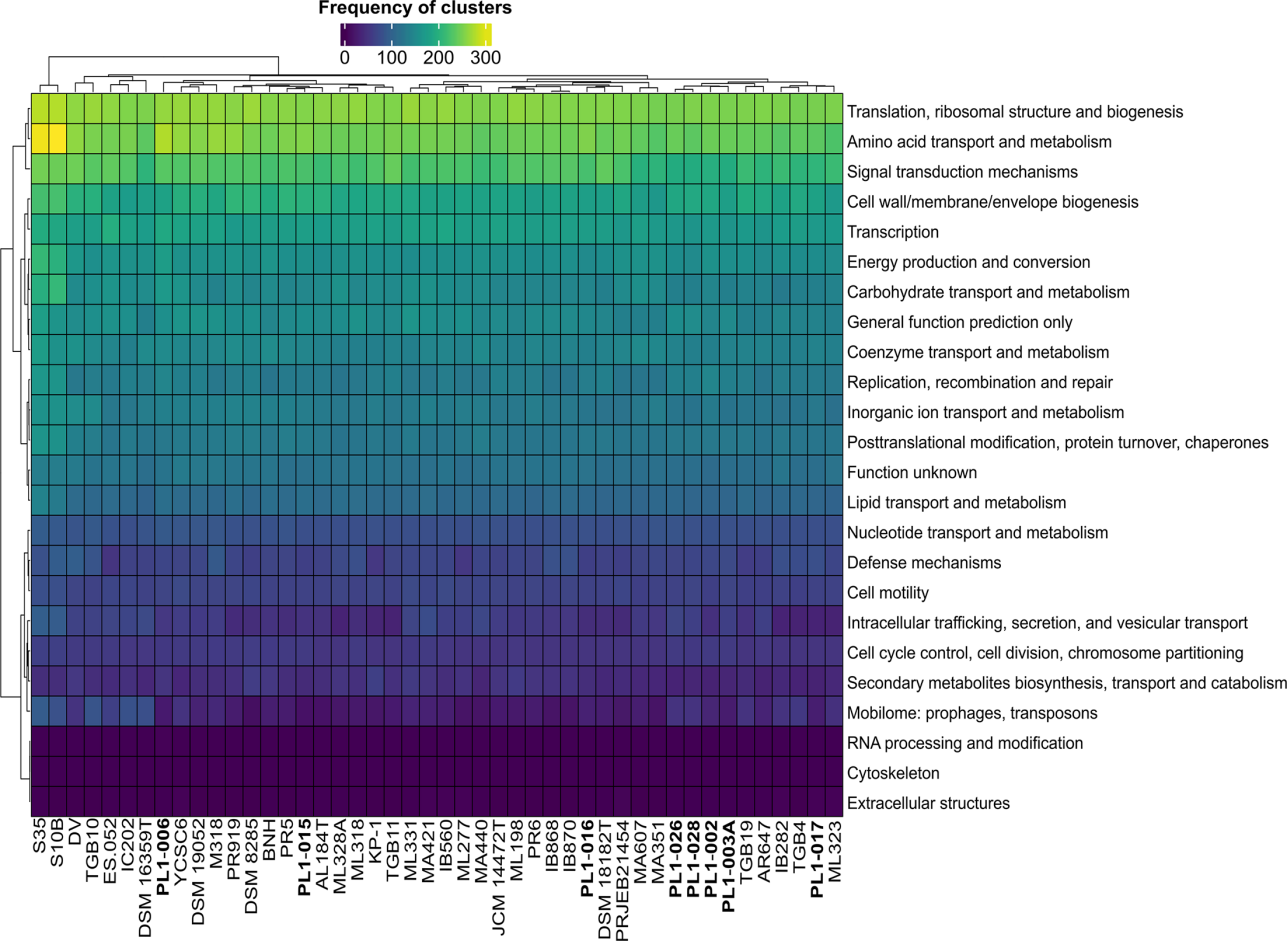
Functional annotation of protein-coding genes present in *Salinivibrio* genomes based on frequency of COG20 categories. The colour indicates the level of abundance. A matrix of the frequency of COG20 categories was exported via Anvi'o and was visualized by the ComplexHeatmap package in R-Studio with Euclidean clustering. The resulting heatmap was edited with Inkscape (https://inkscape.org/) to improve font size.

CDS were assigned and mapped into 24 functional COG20 categories across all 46 genomes (Fig. 6 and Table S1). The most abundant categories were translation, ribosomal structure and biogenesis (J, mean 256 genes per genome), followed by amino acid transport and metabolism (E, 250) and signal transduction mechanisms (T, 222). The high proportion of translation-related genes is notable, as ribosome and elongation factor concentrations are known rate-limiting factors for growth kinetics in closely related Vibrionaceae, such as *Vibrio natriegens* [85,86]. This suggests that *Salinivibrio* may achieve rapid growth rates, potentially contributing to both its ecological success in hypersaline environments and its suitability as a biotechnological chassis organism [6,87]. For the Pearse Lakes strains, no notable differences were observed in COG20 category distribution compared to those of other *Salinivibrio* genomes analysed in this study.

Within the pangenome, core genes were predominantly associated with translation (J), amino acid transport and metabolism (E) and energy production and conversion (C), consistent with selective retention of essential cellular functions, as has been observed for core genomes within the closely related genus *Vibrio* [88] (Table S1). In contrast, dispensable genes were largely composed of mobile genetic elements (prophages and transposons, X), defence mechanisms (V), signal transduction (T) and carbohydrate transport and metabolism (G), whereas singletons were dominated by mobilome (X), defence (V), replication and recombination (L) and cell wall, membrane and envelope biogenesis (M) functions (Table S1). The distribution of these COG categories within the accessory genome, rather than in the core, indicates that the variable gene pool is functionally non-random. Mobile genetic elements offer a plausible mechanism for the recent gene gain and loss detected by Panstripe analysis [29,30, 88]. The prevalence of cell-envelope, defence and environmental-sensing functions is consistent with a role in niche-specific adaptation, as reported for closely related *Vibrio* [88]. These functions, which support membrane adjustment and environmental response, would be advantageous in the fluctuating hypersaline conditions from which the Pearse Lakes strains were isolated, where persistence depends on tolerating a wide range of salinities. Further specific metabolic examples, including pathways relevant to osmotic adaptation, are examined in the following section.

#### KEGG pathway completion analysis for *Salinivibrio* spp.

To characterize the metabolic functions distributed across the *Salinivibrio* core and accessory genome, KEGG module analysis of the 46 genomes identified 9,564 modules, of which 4,298 were complete (>75% KOs present). Central carbohydrate metabolism modules, including the Embden–Meyerhof pathway, pyruvate oxidation, the tricarboxylic acid (TCA) cycle, gluconeogenesis, the pentose phosphate pathway and the Entner–Doudoroff pathway, were complete in all analysed genomes (Table S1).

Energy metabolism pathways revealed several features relevant to halophilic adaptation. The sulphate-sulphur assimilation module (M00616) and assimilatory sulphate reduction pathway (M00176) were complete across nearly all genomes, apart from *S. proteolyticus* DSM 19052^T^, *S. kushneri* TGB19 and *S. sharmensis* DSM 18182^T^ [89] (Table S1). The conservation of these sulphate assimilation pathways within the Pearse Lakes strains is consistent with the high sulphate concentration measured at the sampling site (see hydrochemical analysis). Among carbon fixation modules annotated by KEGG, the reductive pentose phosphate cycle (Calvin cycle) modules M00165, M00166 and M00167 were scored as complete in all genomes. However, detailed analysis of the annotated genes indicated that glycerate 3-phosphate, a key intermediate, is more likely to be produced via glyoxylate and dicarboxylate metabolism rather than carbon fixation, consistent with previous findings in other halophilic micro-organisms [90,91].

**Fig. 7.**
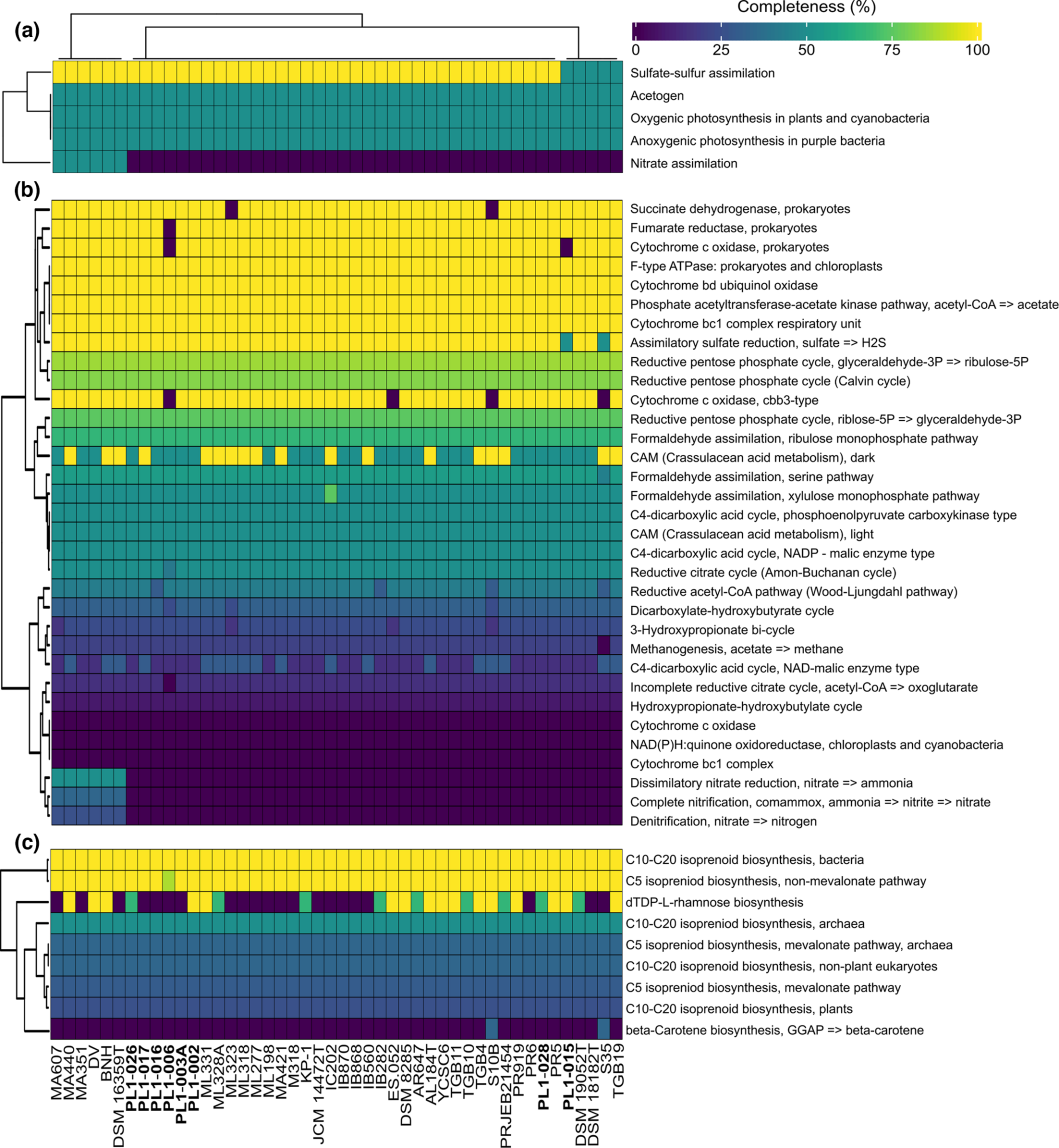
Estimated completeness for KEGG metabolism pathways of interest presented in *Salinivibrio* genomes based on completion of pathways. The colour indicates the level of completeness. Matrix of the completeness of KEGG pathways (defined as completed when >75% KOs are presented within the pathway) was exported via Anvi'o and was visualized by ComplexHeatmap in R-Studio with Euclidean clustering. The KEGG subcategories were mapped as follows: (**a**) module sets, (**b**) energy pathways and (**c**) biosynthesis of terpenoids and polyketides. The resulting heatmaps were edited with Inkscape to correct the font size.

Notably, 18 genomes, including *S. kushneri* PL1-017, contained a complete module for dark crassulacean acid metabolism (CAM) (M00168), with genes encoding malate dehydrogenase (MDH2, K00026) and phosphoenolpyruvate carboxylase (ppc, K01595) detected (Fig. 7b). Rather than indicating autotrophic carbon fixation, the presence of ppc and MDH2 more likely reflects anaplerotic CO_2_ assimilation, in which ppc replenishes oxaloacetate for the TCA cycle and supports central metabolic flexibility. Consistent with an anaplerotic rather than an autotrophic role, phosphoenolpyruvate carboxykinase characterized from *S. costicola* (as *Vibrio costicola*) favours phosphoenolpyruvate synthesis at physiological salt concentrations, with CO_2_ fixation activity maximal only at very low salt [92]. The variable presence of this module across the genus, therefore, points to strain-level differences in central carbon metabolism rather than a capacity for net carbon fixation.

Analysis of terpenoid and polyketide biosynthesis modules identified complete pathways for C5 and C10–C20 isoprenoid biosynthesis in all strains (Fig. 7c). Additionally, 16 strains, including *S. costicola* PL1-002 and *S. kushneri* PL1-015, possessed a complete dTDP-l-rhamnose biosynthesis pathway (M00793). l-Rhamnose is commonly present in the O-antigens of Gram-negative bacteria and has been shown to play a role in high-salt adaptation, as its deletion causes flocculation in other moderate halophiles [93,94]. The variable presence of this pathway is consistent with the enrichment of cell-envelope biogenesis functions in the accessory genome described above, providing a specific example of envelope-associated variation with a plausible role in osmotic adaptation. For the Pearse Lakes strains, no significant differences in KEGG module completeness were observed compared to other *Salinivibrio* genomes. However, clustering differences were apparent between *S. kushneri* and *S. costicola* strains, with *S. kushneri* PL1-017 notably the only Pearse Lakes isolate with a complete dark CAM module (Fig. 7), potentially reflecting phenotypic variability between species isolated from the same environment [95]. Having established the broader metabolic capabilities of *Salinivibrio*, we next examined the presence of genes directly involved in PHA biosynthesis to analyse a core genome pathway.

### PHA biosynthesis and degradation pathways

#### Complete PHA biosynthesis pathway

**Fig. 8.**
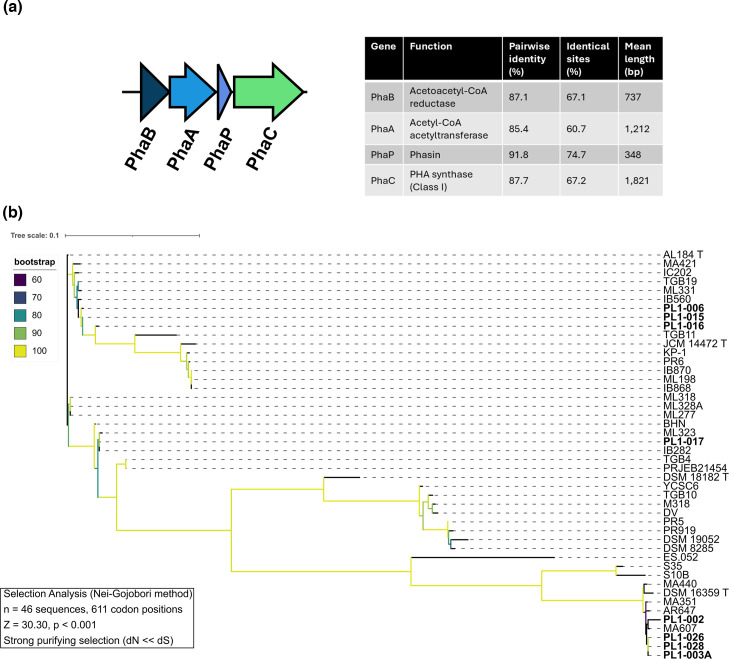
PHA biosynthesis pathway conservation and PhaC phylogenetic analysis in *Salinivibrio*. (**a**) Synteny and conservation of the PHA biosynthesis operon (*phaB*-*phaA*-*phaP*-*phaC*) across 46 *Salinivibrio* genomes. The table shows pairwise amino acid identity, the number of identical sites and the mean gene length. All genes display high conservation (85.4–91.8% identity). (**b**) Maximum likelihood phylogenetic tree of PhaC from protein-guided codon-aligned sequences (611 positions) using IQ-TREE (TNe+G4 model). Branch colours indicate ultrafast bootstrap support (1,000 replicates). Selection analysis demonstrates strong purifying selection (Z=30.30, *P*<0.001), indicating PhaC is under functional constraint across *Salinivibrio* species. Text labels and layout were edited with Inkscape.

The complete PHA biosynthesis operon (*phaB-phaA-phaP-phaC*) was identified in all 46 Salinivibrio genomes as part of the core genomes (Fig. 8a and Table S1). Gene neighbourhood analysis revealed a conserved operon arrangement across all strains, with genes consistently ordered as *phaB* (acetoacetyl-CoA reductase), *phaA* (acetyl-CoA acetyltransferase), *phaP* (phasin) and *phaC* (PHA synthase class I) (Fig. 8a). Pairwise amino acid identity ranged from 84.5 to 91.8% across the four genes, with *phaP* showing the highest conservation (91.8%) (Fig. 8a). The identification of the complete biosynthetic operon extends previous genomic and experimental identification in *Salinivibrio* [6,87, 96, 97], demonstrating that the genus possesses all the enzymes necessary for *de novo* PHA synthesis from central metabolic intermediates. Selection pressure on PhaC was assessed using the codon-based Z-test of purifying selection (Nei-Gojobori method [62]) in mega 11. Analysis of 46 PhaC sequences revealed strong evidence for purifying selection (Z=30.30, *P*<0.001), indicating that non-synonymous substitutions are selectively constrained relative to synonymous substitutions (Fig. 8b). This pattern of strong functional constraint, combined with high sequence conservation across the operon (85.4–91.8% pairwise identity), is consistent with PHA biosynthesis being an essential, selectively maintained function within *Salinivibrio*.

#### Putative PHA degradation pathway

Catabolism of PHA typically involves depolymerization into monomers via PHA depolymerase (PhaZ EC 3.1.1.75 and EC 3.1.1.76) and PHA oligomer hydrolase (PhaY EC 3.1.1.22) [64,98]. No close orthologs of *phaZ* or *phaY* were found in the *Salinivibrio* genomes analysed in this study, and a previous study reported that they may lack PHA degradation capacity [13]. The biosynthesis PHA operon, however, is part of the core genome and is conserved under strong purifying selection. In a genome with ongoing gene gain and loss, the retention of a biosynthetic pathway with no apparent way to recover the stored polymer is unexpected.

**Fig. 9.**
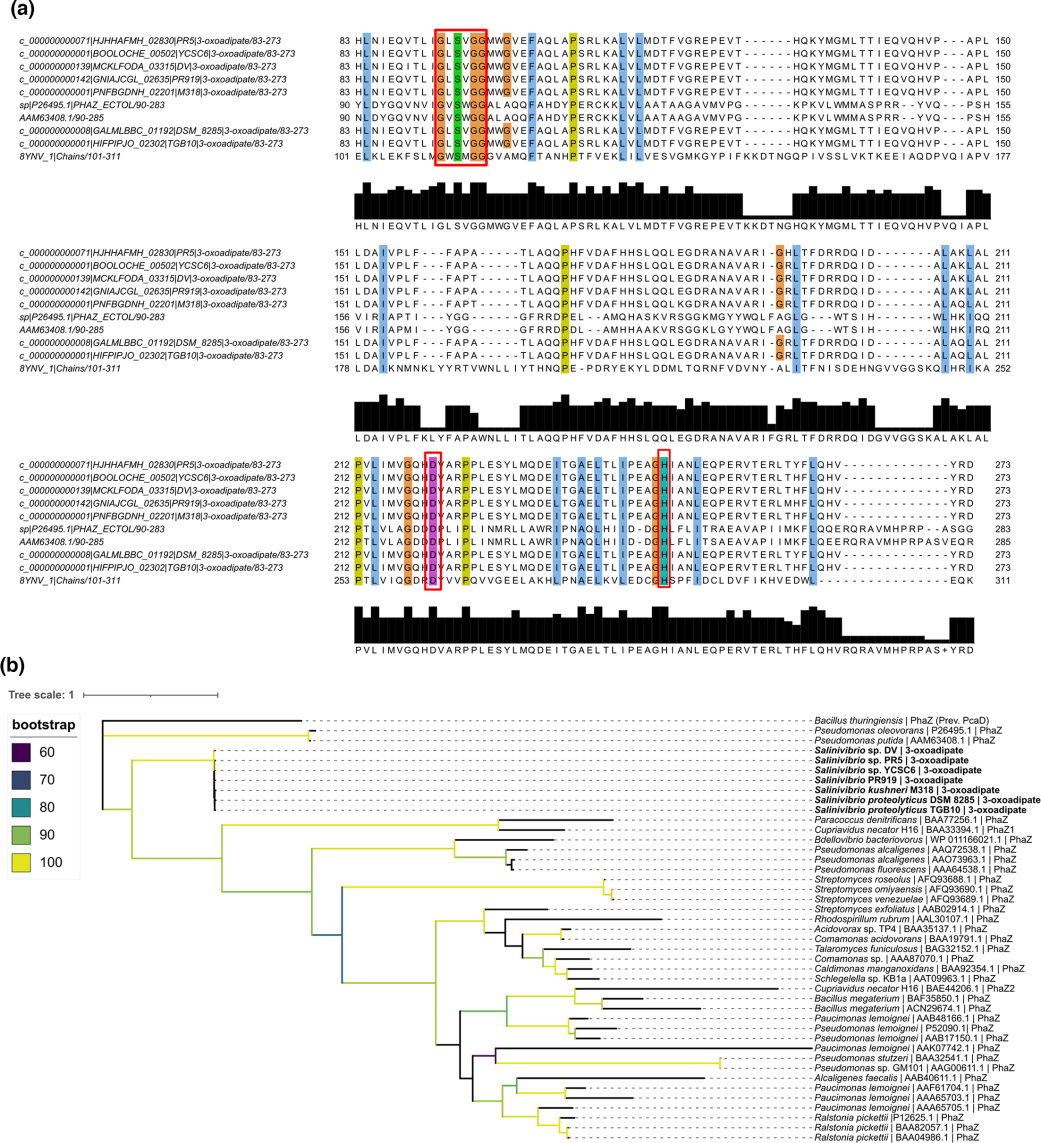
Putative PHA depolymerases in *Salinivibrio* contain conserved catalytic machinery and are grouped with characterized PhaZ. (**a**) Multiple sequence alignment showing three catalytic regions: the GXSXGG lipase box with catalytic serine, a conserved aspartate and a conserved histidine, completing the Ser-His-Asp triad. *Salinivibrio* candidates retain the complete catalytic machinery despite 18–22% overall identity with the reference PhaZ. (**b**) Maximum likelihood phylogeny (IQ-TREE, LG+F+R8 model, 1,000 bootstrap replicates) showing *Salinivibrio* sequences form a monophyletic clade sister to characterized PhaZ with long branch lengths indicating divergence. Branch colours show bootstrap support (yellow: 100%, green: >90%, blue: <70%, purple:<60%). All *Salinivibrio* candidates are annotated as ‘3-oxoadipate enol-lactonase 2’ (K01055).

An HMM profile built from 895 γ-proteobacterial PHA depolymerase genes was used to search all 140,841 protein sequences in the 46 genomes. Seven candidate sequences were identified across seven *Salinivibrio* genomes, each with a complete Ser-His-Asp catalytic triad and the GXSXGG lipase box motif (Fig. 9 and Table S1) [99]. The seven candidates form a single dispensable gene cluster present in seven of the 46 genomes. The putative degradation function, therefore, sits in the accessory genome, whereas the PHA biosynthesis operon is within the core genome. All seven candidates are currently annotated as ‘3-oxoadipate enol-lactonase 2’ (K01055). The same annotation was shown to mask a functional intracellular PHB depolymerase in *Bacillus thuringiensis,* which shared only 4–22% identity with characterized PhaZ [100]. A comparable pattern has been reported in marine Vibrionaceae, where classical PHA depolymerases were absent but divergent lipase-like α/β-hydrolases carrying the same catalytic residues were identified as candidate depolymerases [101].

The *Salinivibrio* candidates show similar sequence divergence (18–22% identity to the reference PhaZs). This mirrors the wider pangenome analysis, where divergent sequences were poorly captured by standard annotation. The same limitation that obscures accessory gene content at the genome scale also concealed a candidate degradation function that a previous annotation-based study reported as absent. These candidates remain putative. Their restriction to a subset of strains, unlike the conserved biosynthesis operon, is consistent either with a strain-specific degradation capacity or with divergent hydrolases of another function. The conserved catalytic residues are consistent with depolymerase activity, but sequence analysis alone cannot confirm function. Confirmation would require biochemical assays of depolymerase activity together with genetic or physiological validation, and analysis of the active site and substrate-binding pocket would test whether the residues are arranged for catalysis.

## Conclusion

This study addresses three hypotheses through the analysis of eight new complete *Salinivibrio* genomes from Pearse Lakes, alongside 38 high-quality public genomes. First, complete genome assemblies from an underexplored hypersaline system revealed genome diversity not captured by previous analyses relying predominantly on draft assemblies, expanding the known *Salinivibrio* pangenome from 5,570 to 8,152 gene clusters. Second, Panstripe analysis provided statistical support for an open pangenome with a significant temporal signal in gene gain and loss events, contrasting with earlier reports of pangenome saturation and indicating that *Salinivibrio* genomes are more dynamic than previously recognized. Third, HMM-based homology searching identified seven putative PHA depolymerase candidates potentially misannotated as 3-oxoadipate enol-lactonases, demonstrating that annotation-dependent approaches can obscure divergent enzyme families in non-model organisms. The universal conservation of the PHA biosynthesis operon under strong purifying selection establishes PHA production as a core metabolic function within *Salinivibrio*. This genomic capacity is supported by experimental studies demonstrating efficient PHA accumulation in *Salinivibrio* strains, including poly-3-hydroxybutyrate titres exceeding 100 g l^−1^ at near-theoretical yield and copolymer production under non-sterile, high-salt conditions [6,87, 96, 97, 102], reinforcing the potential of the genus as a halophilic chassis for bioplastic production. The identification of putative degradation enzymes in a subset of strains, if validated, would indicate that some *Salinivibrio* species also possess the capacity to degrade PHA, with implications for understanding PHA metabolism in halophilic bacteria and for developing enzymatic approaches to bioplastic processing under saline conditions.

## Supplementary material

10.1099/mgen.0.001786 Fig. S1.

10.1099/mgen.0.001786 Table S1.

## Abbreviations

ANI: average nucleotide identity
BUSCO: Benchmarking Universal Single-Copy Orthologs
CAM: crassulacean acid metabolism
CDS: coding DNA sequence
COG: clusters of orthologous group
CTAB: cetyltrimethylammonium bromide
dDDH: digital DNA–DNA hybridization
HMM: Hidden Markov Model
IM: isolation medium
KEGG: Kyoto Encyclopedia of Genes and Genomes
LB: lysogeny broth
MCL: Markov cluster algorithm
MDH2: malate dehydrogenase
MLSA: multi-locus sequence analysis
ONT: Oxford Nanopore Technology
PHA: polyhydroxyalkanoate
ppc: phosphoenolpyruvate carboxylase
TCA cycle: tricarboxylic acid cycle
WA: Western Australia
