# Supplementary figures and images for: Refining Salinivibrio pangenome dynamics and biotechnological potential through comparative analysis
Source: Microb Genom. 2026 Jul 20;12(7):001786. doi: 10.1099/mgen.0.001786 (PMC13384114; doi:10.1099/mgen.0.001786)

Tree scale: 0.01

bootstrap

75

81.25

87.5

93.75

100

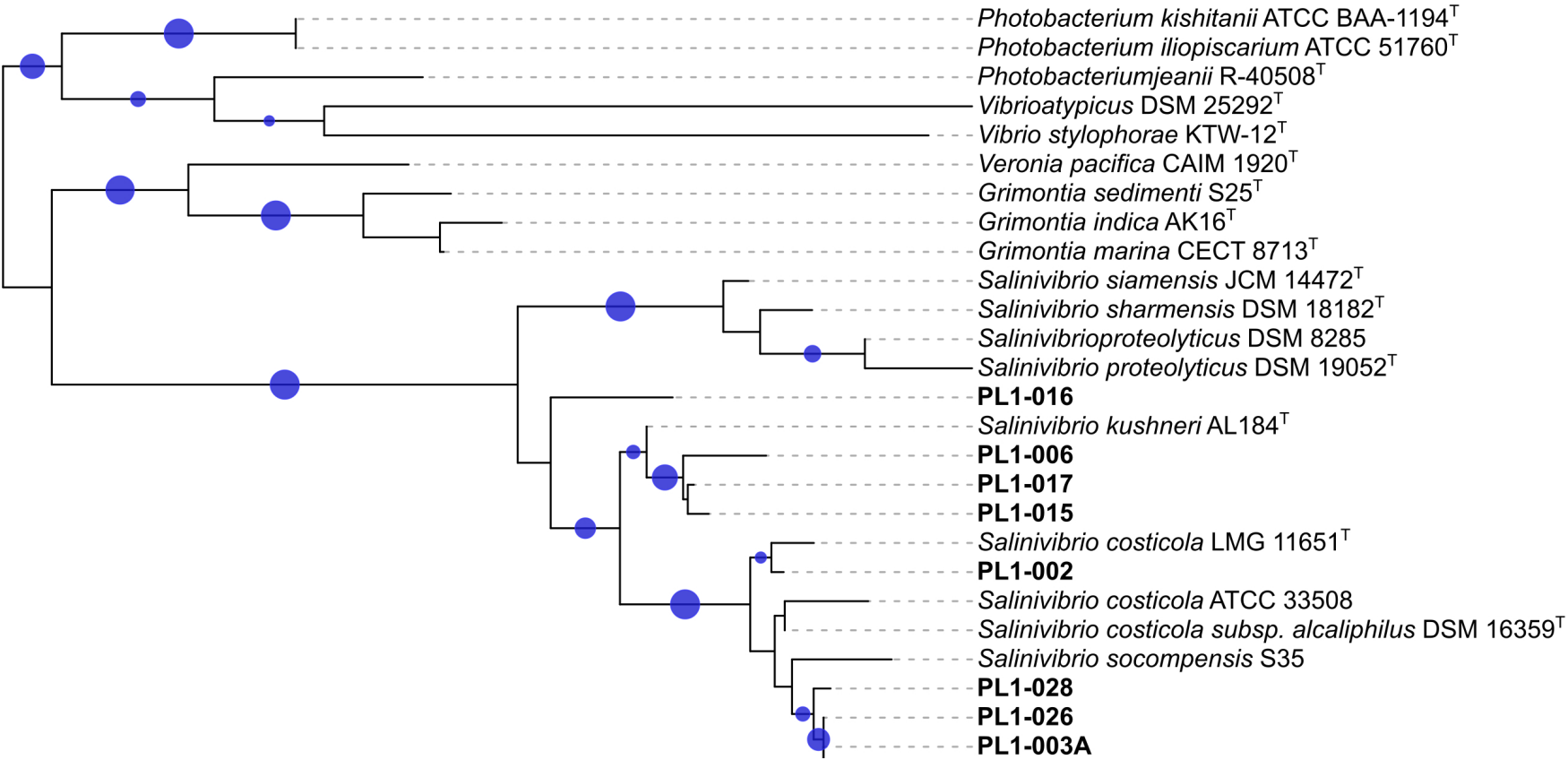

Supplement: Fig. S1. [file mgen-12-01786-s001.pdf]
